# Psychiatric Presentations of Hypoglycemia in Adolescents With Type 1 Diabetes: A Diagnostic Pitfall

**DOI:** 10.1002/ccr3.72659

**Published:** 2026-05-03

**Authors:** Chukwuka Elendu, Excel N. Victor‐Anozie

**Affiliations:** ^1^ Federal University Teaching Hospital Owerri Nigeria; ^2^ Federal Medical Centre Asaba Nigeria

**Keywords:** adolescents, fluoxetine, hypoglycemia, psychiatric symptoms, type 1 diabetes

## Abstract

Hypoglycemia in adolescents with type 1 diabetes can mimic primary psychiatric disorders and lead to misdiagnosis. Clinicians should routinely exclude hypoglycemia in acute behavioral changes, as psychotropic medications such as fluoxetine may worsen unrecognized episodes in the absence of adequate glycemic monitoring.

Dear Editor,

We read with great interest the recently published case report titled “Psychiatric Manifestations of Hypoglycemia in an Adolescent: A Case Report” [1], describing a 17‐year‐old with type 1 diabetes mellitus whose recurrent behavioral disturbances were initially misdiagnosed as a primary psychiatric disorder and worsened after fluoxetine initiation before being attributed to hypoglycemia. We commend the authors for highlighting this important and underrecognized interface between endocrinology and psychiatry, as well as the potential for psychotropic medications to influence glycemic stability.

Hypoglycemia is a well‐recognized complication of insulin therapy in adolescents with type 1 diabetes and is typically defined as plasma glucose < 70 mg/dL (3.9 mmol/L), with clinically significant levels < 54 mg/dL (3.0 mmol/L) [1]. Owing to the brain's dependence on glucose, neuroglycopenia can manifest as confusion, agitation, hallucinations, and abnormal behavior—features that may predominate in adolescents and mimic primary psychiatric disorders [2, 3, 4]. Consequently, hypoglycemia may be overlooked during psychiatric evaluation, particularly when psychosocial stressors are present. In this case, factors such as recent diagnosis and family stress may have contributed to diagnostic anchoring and premature closure, emphasizing the need to exclude reversible medical causes before assigning psychiatric diagnoses [5, 6]. However, it is also important to acknowledge the potential for retrospective or attribution bias in interpreting this sequence of events, as the identification of hypoglycemia after the fact may influence how earlier clinical decisions are appraised.

An important feature of this report is the temporal association between fluoxetine initiation and worsening hypoglycemic episodes. Although selective serotonin reuptake inhibitors are often considered metabolically neutral, evidence suggests that fluoxetine may increase insulin sensitivity and peripheral glucose uptake, thereby increasing hypoglycemia risk in insulin‐treated patients [7, 8, 9, 10, 11, 12, 13, 14]. However, current evidence supports an association rather than a direct causal relationship, and this distinction is important in interpreting the findings of this case. While the absence of insulin dose adjustment strengthens the plausibility of a contributory role, fluoxetine should be considered a potential precipitating factor rather than a definitive cause. Importantly, the absence of objective glycemic data, such as self‐monitoring of blood glucose (SMBG) or continuous glucose monitoring (CGM) trends, limits the strength of causal inferences regarding the relationship between fluoxetine initiation and hypoglycemic episodes in this case. Notably, although fluoxetine may independently cause neuropsychiatric symptoms, the rapid resolution of symptoms following carbohydrate intake strongly supports hypoglycemia as the primary driver [15, 16, 17].

This case also highlights challenges in adolescent diabetes care. During the early post‐diagnosis ‘honeymoon’ phase, insulin requirements often decline, and failure to adjust dosing increases hypoglycemia risk [18]. However, insulin requirements during this phase are highly variable, and emerging evidence suggests that total daily doses around 0.9 ± 0.3 IU/kg/day may be observed in newly diagnosed patients, with only a subset demonstrating a clear honeymoon phase [19]. While the insulin dosing in this case may have contributed to hypoglycemia, caution is therefore warranted in labeling a total daily dose of approximately 1 IU/kg/day as excessive in isolation, particularly without longitudinal glycemic data or evidence of endogenous insulin recovery. In this context, several primary contributors likely played a more central role in precipitating hypoglycemia, including inadequate glucose monitoring, suboptimal insulin regimen characteristics, and lack of timely dose adjustment. Limited access to glucose monitoring, as described, further exacerbates this risk—particularly in resource‐constrained settings, as it delays detection of falling glucose levels and limits opportunities for prompt intervention. Furthermore, the use of intermediate‐acting insulin such as NPH is well known to predispose to nocturnal hypoglycemia due to its peak action profile [18], which may align with early‐morning neuroglycopenic symptoms observed in this patient. This pharmacokinetic profile—characterized by a pronounced peak action occurring several hours after evening administration—can result in a mismatch between insulin activity and nocturnal glucose availability, thereby increasing the risk of unrecognized overnight hypoglycemia and early‐morning neuroglycopenic manifestations. Moreover, diabetes distress may contribute to behavioral symptoms and complicate assessment, underscoring the importance of integrating psychosocial screening into routine care [20].

The sociocultural context, including attribution of symptoms to spiritual causes, underscores the need for culturally sensitive caregiver education on recognizing and managing hypoglycemia. Delayed recognition can result in severe complications, including seizures and neurological injury [2].

At a systems level, this case illustrates the risks of fragmented care. Despite a known diabetes diagnosis, blood glucose testing was not performed during initial psychiatric evaluation. Routine glucose assessment should be a minimum standard in patients with diabetes presenting with acute behavioral changes, particularly when symptoms are episodic or improve with food intake. From a practical standpoint, clinicians may adopt a structured approach when evaluating such patients: (1) immediate point‐of‐care blood glucose testing at presentation, (2) assessment of symptom timing in relation to meals and nocturnal periods, (3) review of insulin regimen, including type, dosing, and recent adjustments, and (4) evaluation for potential precipitating factors such as medication changes or limited glucose monitoring. Early recognition and correction of hypoglycemia can prevent unnecessary psychiatric interventions and associated morbidity [6, 9].

In conclusion, hypoglycemia can closely mimic primary psychiatric disorders in adolescents with type 1 diabetes, creating a significant diagnostic pitfall. Clinicians should maintain a high index of suspicion and routinely assess blood glucose in such presentations. Greater interdisciplinary collaboration, improved access to monitoring tools, and caregiver education are essential to prevent misdiagnosis and improve outcomes.

## Author Contributions

**Chukwuka Elendu:** conceptualization, data curation, formal analysis, investigation, methodology, project administration, supervision, validation, visualization, writing – original draft, writing – review and editing. **Excel N. Victor‐Anozie:** validation, visualization, writing – review and editing.

## Funding

The authors have nothing to report.

## Disclosure

The views expressed in this report are solely those of the authors and do not represent the official positions of any affiliated institutions.

## Ethics Statement

The authors have nothing to report.

## Consent

Written informed consent for publication was obtained by the authors of the original report. This letter does not include new patient data.

## Conflicts of Interest

The authors declare no conflicts of interest.

## Data Availability

No datasets were generated or analyzed for this letter.
